# Pedagogical strategies for supporting learning and student well-being in environmentally sustainable healthcare

**DOI:** 10.3389/fmed.2025.1446569

**Published:** 2025-02-27

**Authors:** Nara Jones, Graeme Horton, Michelle Guppy, Georgia Brown, John Boulton

**Affiliations:** ^1^School of Medicine and Public Health, University of Newcastle, Newcastle, NSW, Australia; ^2^School of Medicine, University of Tasmania, Hobart, TAS, Australia; ^3^School of Rural Medicine, University of New England, Armidale, NSW, Australia

**Keywords:** planetary health, health professional education, sustainable healthcare, educational theory, student well-being

## Abstract

Planetary health education needs fresh approaches to engage learners and educators in positive visions and future planning to navigate the societal challenges of climate change. The human health impacts of the climate crisis, environmental degradation and pollution are far-reaching and compounding in nature. International leaders in healthcare are recognizing the time-pressured opportunity to mobilize and motivate colleagues to optimize health outcomes by addressing these issues. Healthcare systems across the globe contribute significantly to ecological footprints through greenhouse gas emissions and consumption of various polluting materials. Therefore, the necessity to prepare future health professionals to identify and manage environmental health conditions in their patients, as well as foster their future role as leaders and advocates in sustainable healthcare is acute. Health education organizations have begun to appreciate this need and have developed learning objectives to guide curricula. In the development and implementation of content on environmentally sustainable healthcare, an important consideration is the affective and moral distress from the confronting and often overwhelming nature of the topic. The main objective in teaching planetary health is to equip learners with the tools and skills to address the relevant health issues in their professional role whilst providing the support necessary for them to accept these harsh realities. The University of Newcastle and University of New England Joint Medical Program's, four-week course in Sustainable Healthcare aims to meet this objective. In this article we discuss how our curriculum utilizes Self-Determination Theory (SDT) and other psychological strategies to support learners' well-being and motivation. SDT explains the need for supporting autonomy, relatedness and competence in the learning environment. Strategies employed to address these include providing students with the opportunity to select discussion topics that they contribute to, maximizing choice of focus for the assessment task, utilizing personal reflections, case-based learning scenarios and incorporating presentations from relatable industry leaders.

## 1 Introduction

As medical students are at risk of emotional distress when they learn about the effects of climate change on individual and population health, their well-being must be seen as a priority in order for them to maintain their motivation to engage with the subject. This is also critical for their understanding that their future professional involvement needs to be guided by moral values. Human health has long been recognized to be dependent on healthy and intact ecosystems, but the environmental determinants of health are now changing in unprecedent ways and rates. Planetary health has emerged in the wake of global events and encompasses climate change, biodiversity loss, pollution and the need for humans to live, work and to maintain their health in ways that do not compromise the ability of future generations to do the same (1).

In order to be fit for purpose as future doctors, medical students need to learn about the changing environmental determinants of health, understand the health impacts of planetary degradation and how to mitigate risk to their patients, practice in a sustainable way and advocate for reducing the environmental impact of the health care system (2). The addition of curricula to prepare medical students for managing the impacts of climate change or how to practice in an environmentally sustainable way is relatively recent. In 2019 an international survey of medical students identified that only 15% of medical schools had begun to include climate change and health into their curriculum (3). In recent years health professional education stakeholders have proposed learning objectives (4) and recommendations to assist in developing planetary health curricula (2, 5, 6). Whilst a number of programs have begun to implement and evaluate curricular interventions in this area (7–17), there has been little consideration as to which educational approaches support student well-being. Climate change is now causing widespread illness, injury and death from direct effects such as extreme weather events and sea level rise, and indirect causes such as crop failure, civil unrest and population displacement (18, 19). Further worsening impacts from climate change are unavoidable, and the likelihood of societal collapse and widespread suffering is increasingly described in the literature (20). The scale of the challenge of working to ensure that humanity lives within the capacity of the planet is such that students can feel overburdened. Uncomfortable feelings in learners can arise as they grapple with complex real-world problems entrenched in politics, exploitation, social disadvantage, and power imbalances (2).

Eco-anxiety, climate change distress and solastalgia are terms to describe negative emotions that relate to concern about the future, the loss that has already occurred, and the adverse consequences of human impact on the environment (21, 22). The psychological response to environmental degradation encompasses a wide range of emotions, especially in the younger generations. In their international study that surveyed young people from ten different countries on climate change distress, Hickman et al. found that of the 10,000 participants, over 50% reported feeling anxious, angry, sad, afraid, powerless, helpless and guilty (21). Furthermore, over 45% of participants felt that their feelings negatively impacted their daily life (21). These emotions originate not only from the concerns of the devastating impacts of environmental degradation, but also from the perceived lack of influence to tackle the enormous task ahead. The majority of power, when it comes to preventing and mitigating the impacts of threats to the planet, is held by governments, dominant industries and large corporations. Not only do individuals perceive they have little influence over these bodies, but there is also a sense that these entities are not adequately addressing the issues at hand. The Hickman et al. study also found that only 30% felt that their governments were taking their concerns about climate change seriously and 58% they felt their governments were betraying them and/or future generations (21). While this international study is the largest quantitative study contributing data to our understanding of emotional distress in young people related to environmental degradation, surveys of young people in Australia and the UK have demonstrated that significant proportions, 89% and 74%, respectively, of young people are worried about the effects of climate change (23, 24). A recent study of over 15,000 young people in the US demonstrated that 85% of participants were at least moderately worried about climate change and it's potential impacts (25). There is also significant concern raised by academics and health professionals regarding the direct and indirect effects on the psychological well-being of young people (26–31). Furthermore, the significant increase in youth led climate activism globally demonstrates the level of concern for the future amongst our youth (29). While the psychological impact of learning about sustainable healthcare on medical students has not been specifically investigated, students within studies evaluating a sustainable healthcare curricula intervention have described learning about climate degradation and the effects on patients as “scary”, highlighting the importance of curricular to create a sense of empowerment to balance the potential distress and reduce the chance of becoming “disillusioned” or “disengaged” (10, 32). Educational stakeholders have recognized that we have a responsibility to consider student well-being and cultivate resilience for learning about this potentially confronting and “sobering” topic (33) and dealing with planetary health related uncertainty (2).

There is much to be gained by educators utilizing psychological theories that optimize human motivation for planetary health education. Implementing learning strategies underpinned by psychology enables knowledge acquisition, while supporting students to develop adaptive coping strategies and capacity to enact change in the face of uncertainty.

## 2 Pedagogical frameworks

Compared to when many of today's educators may have learned about motivation in the last decades of the previous century, we now better understand the basic psychological needs of people which if catered to, can provide conditions for optimal performance, creative problem-solving and conceptual learning (34). Faced with current and future planetary health challenges, professionals need to be optimally motivated and engaged. Self-determination theory, as developed by Deci and Ryan, explains that better outcomes result when a person is autonomously motivated, rather than controlled or incentivized contingent on performance. In other words, there are different types of motivation, and when people are intrinsically interested in an activity, or personally identify the value of doing it, then they will be more energized and effective than if they feel or are forced into doing something, whether due to obtaining rewards and approval or to avoid shame or punishment (35). Social contexts, including educational settings, can support these natural tendencies or thwart them. The provision of the deep needs of people for autonomy, competence, and relatedness allows optimal human flourishing. Autonomy is the need to self-regulate one's experiences; a sense of voluntariness as distinct from independence. A hallmark of autonomy is that one's behaviors are self-endorsed. Competence refers to the need to feel effective and feel mastery when completing tasks. Relatedness is to feel socially connected, feel cared for by others, and contribute to others.

If these needs are satisfied, people are more likely to develop and function effectively and experience wellness. If these needs are thwarted, people will more likely evidence ill-being and non-optimal functioning. Classroom studies have shown that provision of autonomy to students by both teachers and parents has helped students maintain intrinsic motivation (36). Autonomously motivated learning has been found to lead to better educational outcomes, and medical students who have had autonomy-supportive learning have been shown to provide patient care that is more autonomy supportive (37).

Optimizing motivation is part of what is required in delivering planetary health education, but equipping learners with the emotional tools to manage the knowledge that they acquire is just as important. Climate change distress may be eased with judicious use of adaptive coping. Seth and colleagues have helpfully explained that such strategies comprise:

- Emotion-focused coping which addresses the feelings associated with climate change.
- Problem-focused coping which leads to behavioral responses.
- Meaning-focused coping that builds on hope and one's values (38).

The Australian Psychological Society outlines examples of adaptive coping strategies in their “Coping with change distress” resource. These include: taking a break from the 24/7 news cycle, taking specific action that is within one's own control to reduce their own carbon footprint, and prioritizing issues that are the most important, recognizing that no-one can do everything (39).

A third theoretical framework that is applicable to this area concerns taking action that is based on what is important to us. Acceptance and Commitment Therapy (ACT) has been identified as particularly suited to climate change distress with confronting concepts that cannot be refuted, highlighting the benefits of values-guided action. ACT provides guidance on how to reduce the impact and consequences of uncomfortable feelings and thoughts while planning actions to build a rich and meaningful life (40). While ACT is designed for clinical application, educators can apply some of these principles in guiding students to consider what is important and meaningful to them, and then to use these values to develop plans for actions that will enrich and enhance their lives and the world around them.

## 3 Learning environment

To demonstrate how principles of adaptive coping, self-determination theory and values-based action can be applied in planetary health curricula, we will describe the development of the sustainable healthcare course of the Joint Medical Program of the University of Newcastle and the University of New England (JMP). This course has been based upon the learning objectives of the Medical Deans Australia and New Zealand (MDANZ) Working Group on climate change and health. In 2017, a group of educators came together to develop learning objectives which were distributed to all medical programs in Australia and New Zealand as a resource that could be used to incorporate planetary health learning objectives in their curricula (41). The learning objectives were also published in the Medical Journal of Australia (4). The specific learning objects for the JMP course can be found in Table 1.

**Table 1:** Learning objects JMP sustainable healthcare course.

**Week 1—module 1: science and scholarship**
Outline the dependence of human health on global and local ecological systems which supply clean air, clean water and nutritious food and the Earth systems that provide a stable climate.
Discuss the contribution of human activity to global and local environmental changes such as climate change, and biodiversity loss and resource depletion in land and marine environments.
Describe the mechanisms by which human health is affected by environmental change, e.g. exposure to extreme weather, change in disease vectors, migration and decreasing food and water security.
Explain how the health impacts of environmental change are distributed unequally within and between populations and the disparity between those most responsible and those most affected by change.
**Week 2—module 2: clinical practice**
Prevent, diagnose and treat the adverse health effects attributed to climate change and environmental causes.
Propose ways to practice medicine sustainably by considering what models of care could reduce the environmental impact of best practice care and service delivery to patients.
Propose ways to practice medicine sustainably by considering the environmental impact of medications and other treatments in prescribing decisions.
Identify the vulnerabilities of health services and health facilities to climate change and extreme weather events and how these risks can be minimized and prepared for.
Explain the concept of 'health co-benefits' by considering how lifestyle choices can promote both patient well-being and a healthy environment.
**Week 3 and 4—module 3: health and society, professionalism**
**and leadership**
Identify the role of health care professionals in advocating for policies and infrastructure that promote the availability, accessibility and uptake of healthy and environmentally sustainable behaviors.
Describe features of a health-promoting local environment, in community and healthcare settings, to include access to green spaces, clean air, and an active travel infrastructure.
Explain how trends in climate change may affect capacity to provide healthcare in the future.
Explain the contribution of the Sustainable Development Goals to addressing the socio-economic and environmental determinants of health.
Identify ways to improve the environmental sustainability of healthcare systems by reducing the carbon footprint through individual practice, health service management and the design of care systems.

Learning objectives derived from the MDANZ Working Group on climate change and health (4).

The four-week sustainable healthcare course commenced in 2021 as core learning for Year 3 of the JMP. The JMP is a 5-year program, of which the first two years are primarily classroom based and include a brief two-day General Practice (GP) placement. Year 3 of the program includes GP, hospital and classroom-based rotations. The sustainable healthcare course is conducted several times per academic year, with approximately one eighth of the student cohort completing their course at any one point. Therefore, students' prior exposure to clinical placements varies depending on when in the year their sustainable healthcare course occurs. A breakdown of JMP Cohort Demographics can be found in Table 2.

**Table 2:** Cohort Demographics of three year groups of Joint Medical Program Students who had completed the Sustainable Healthcare course by 2024.

**Age Range (years)**	**18–24**	**25–30**	**31–39**	**40–50**
	352 (60%)	174 (30%)	41 (7%)	15 (3%)
**Gender**	**Male**	**Female**	**Non-binary**	
	281 (48.3%)	299 (51.4%)	2 (0.3%)	
**Total 582**				

In developing the content, we were cognizant of balancing the emotional load for students whilst equipping them with knowledge and skills in this field. The course comprises three modules, each completed over one week. The module's content aligns with both the MDANZ Working Group learning objectives, and three of the four Australian Medical Council (AMC) domains for graduate outcomes; Science and Scholarship, Clinical Practice and Health and Society (42, 43). Throughout the program, we present a range of resources from different sectors, aimed at a variety of audiences for students to reflect upon how best to communicate about climate change and planetary health. Each module centers around carefully curated shared resources which lead to tasks for students to complete. These relate to real-world problem solving within Australian and international healthcare settings, with an emphasis on the Australian healthcare setting. Each task incorporates opportunities for student reflection. Once per week, learners and their academic facilitator engage in a small group tutorial, where they discuss their reflections and engage in case-based learning. The final week of the four-week course is set aside for preparing and delivering their final assessment presentations.

### 3.1 Module 1 relates to science and scholarship

This Module begins with the tenet that human health is fundamentally dependent on healthy ecosystems. As part of this module, students are tasked with watching an introductory presentation about planetary health and making a note of what made the greatest impression on them. This is when students introduced themselves to each other within the group.

Students consider the effects of climate change in Australia as depicted in a video by the Bureau of Meteorology. The task is to consider what climate change impacts have been most apparent where each of the students are living or regard as home. Two articles are presented that utilized air quality data to determine the burden of disease, mortality and economic impact of the 2019–2020 bushfires in Australia (44, 45). This presents a chance for students to consider how environmental health data and modeling can be used to highlight impact contemporaneously, as well as how socioeconomic and health impacts of environmental change are unevenly distributed within and between populations.

In recognition that reading and thinking about climate change and other large scale environmental threats can be confronting, and sometimes distressing, students are referred to a resource “Coping with Climate Change Distress” developed by The Australian Psychological Society (39). Students are directed to think about how these techniques could be useful to themselves and others, and discuss which of the “recommended activities” in the resource resonate with them.

### 3.2 Module 2 relates to clinical practice

Within this Module students explore the need to adapt clinical practice to manage the health impacts of climate change, the ecological footprint of the healthcare system and the concept of “health co-benefits”.

Students learn that health services and facilities are vulnerable to climate change and extreme weather events. They are directed to the World Health Organization “WHO Guidance for Climate Resilient and Environmentally Sustainable Health Care Facilities” to consider how the outlined consequences of climate change for health care facilities could apply to their region or a community of practice (46). The task is to bring to the tutorial an idea for a way of safeguarding the health care facility and/or its staff from this consequence of climate change and whom they could approach to have this implemented.

The Module examines how healthcare itself has a large ecological footprint. Students are directed to read an article that argues that health professionals have a role in advocating for reducing the environmental footprint of healthcare (47). In their assigned task, students think of an example of how they became more aware of their personal environmental impact and how this led to behavioral change. Students watch a presentation by the Local Health District's Executive Director of Infrastructure and Planning on leading and managing change in healthcare, such as new models of care to reduce environmental impact and achieve carbon neutrality whist maintaining best practice care for patients.

Additionally, the idea of sustainable medical clinical decision-making is introduced. Sustainability in medication prescribing is now mainstream in the UK's NHS (48), and explained in an article from the UK National Institute for Health and Care Excellence (49, 50). Students reflect on how they can apply these principles in patient consultations about respiratory inhalers, considering factors of patient preference, compliance and medication safety. The final component of this module introduces students to “health co-benefits”, highlighting that lifestyle choices can promote both patient well-being and a healthy environment. Students interact with television media from Australia's national broadcaster that engages viewers in understanding the carbon footprint of different foods. Students also contemplate the best way for patients to receive information about the environmental impact of medicines, diet and lifestyle advice.

### 3.3 Module 3 relates to advocacy

In Module 3, students consider opportunities for doctors and other health professionals to advocate for improved health of patients and the environment. Examples given include:

- Patient education about healthy lifestyle choices (diet, exercise, transport options) that have environmental co-benefits,
- Writing and releasing reports, contributing to public and academic forums,
- Position statements from professional colleges,
- Submissions to government committees of enquiry and lobbying members of parliament, and
- Media comment, interviews and advertisements.

Students read the report produced by Doctors for the Environment, Australia entitled “Net zero carbon emissions: responsibilities, pathways and opportunities for Australia's healthcare sector” and are tasked with discussing how effectively this report conveys its message and ways that it could be improved (51).

Students watch two interviews with one student and one clinician leader in the field of environmental advocacy and sustainable healthcare practice, and a TED talk by a representative of Doctors for the Environment. The task is for students to share perspectives on leadership qualities that they observe in watching these advocates. Students engage with a multi-media comedic article and reflect on how humor has a place in communication about planetary health issues (52).

Lastly, the Module highlights how planetary health relates to the United Nations' Sustainable Development Goals (SDGs). Multiple nations, including Australia, have signed up to the SDGs as a way of addressing socio-economic and environmental determinants of health, and these are pertinent to planetary health on an international scale. ‘Health equity' is considered using resources of the World Health Organization and a presentation by Professor Michael Marmot on the socioeconomic determinants of health inequity (53). The task is to reflect on one of these SDGs and consider how health professional advocacy and action could enable progress toward this SDG in a particular community. This activity brings together a range of sustainability challenges, allowing students to consider a multidisciplinary lens for problem solving and to explore an interest area in more depth.

### 3.4 Tutorial case-based discussions

Throughout the course, small groups of 8–12 students meet weekly with a tutor for 90-minutes to review the learning from each module. The assigned reflective tasks form the basis of student led discussions, providing structure to the tutorials. Each week we give students the opportunity to write their name next to the two or three tasks for which they would like to lead the discussion and contribute what they have learned. This provides a reliable way of starting the group discussion and avoids what can otherwise be awkward pauses or students feeling put on the spot.

In addition, for each module the group navigates through a clinical case that is related to planetary health. Each case discussion includes aspects of altered illness patterns from climate change, as well as key tenets of clinical practice, ethics and the social determinants of healthcare.

The case study for Module 1 is an older woman who presents with a bushfire related exacerbation of asthma. As well as the clinical diagnosis and management of her condition, students consider issues including shared decision-making about the patient returning to her home, the risks that this may pose to her health, and longer-term worries about the prospect of fires becoming more frequent with climate change.

The case study for Module 2 is an older man who has a syncopal episode in his backyard during a heatwave. Students need to consider how to assess syncope, how to classify heat related illnesses, and both the acute and longer-term management priorities.

In Module 3 students discuss a young student who presents with a history of fever, rash and joint pains. Her travel history requires consideration of zoonoses including Ross River virus and dengue fever. Students discuss how environmental factors influence vector population breeding habitats and patterns, and the subsequent impact on disease incidence and geographical spread of communicable disease outbreaks. They explore the role of the public health units in prevention and response to such changes.

### 3.5 Assessment

In the assessment for this course students are required to submit a pre-recorded ten-minute presentation in week four of the course. All presentations are viewed in a seminar, with questions and student-led discussion following each presentation. Students need to choose three learning objectives to address in their presentation - one of the learning objectives from Module 1, one from Module 2, and one from Module 3. Since these are quite general, students are able to focus their presentation on a particular topic that is of most interest to them.

## 4 Experience of course delivery

This program has now been delivered to over 700 medical students since 2021. As this was a new program with delivery being online and utilizing zoom tutorials due to the COVID-19 pandemic, we were keen to understand how students engaged with the material and whether they could achieve the learning outcomes in a remote format. Following overall positive feedback, we continued to deliver this material online and this provided students with more choice of how and where they spend this month-long course.

The modules gave students the opportunity to explore the diversity of resources in as much or little depth as they liked. Being able to choose their own assignment topic and research in depth gave them the freedom to explore what was of interest to them, and creating a video was an engaging way to learn. For example, students' presentations have addressed topics as varied as anesthetic gases, microplastics, air pollution and active transport (Table 3). Some students drew on their own experiences living in a particular location, and personal experiences of climate impacts such as bushfires, floods and droughts. We received early feedback that students needed more direction about how much to cover given the range of areas was broad. We responded by ensuring that students were comfortable with the expectations after each tutorial and clear about the idea being to break complex information down into teachable moments for the other group members.

**Table 3:** Examples of assessment topics.

**Topic area**	**Specific examples**
Climate event or environmental hazard related to location	Sea level rise in Pacific Islands, Bangladesh and Japan, Water security for remote Indigenous communities in Australia, tuberculosis in India, malaria in Papua New Guinea, dengue fever in Singapore, Flooding in Malaysia, bushfires in Australia, heat waves in Japan
Environmental issue	Micro-plastics, pharmaceutical manufacture and waste, funerary practices, sanitation in healthcare, green spaces in hospitals
Clinical specialty	Environmental footprint of radiology, renal health with emphasis on dialysis, telehealth, surgical waste, anesthetic gases, public health initiatives

The reflective tasks, and subsequent discussions, were observed to have provided an opportunity for students to connect over common experiences, challenges and goals. The case-based learning applied the concepts of planetary health into a clinically relevant context. Students had many opportunities to link the cases to recent climate events, and relate them to their personal experiences of local bushfires, major flooding events and outbreaks of zoonoses such as Japanese encephalitis in previously unreported regions.

Each year, students have been asked to complete an anonymous evaluation about the sustainable healthcare course. Since its commencement in 2021, 51.1%, 39.7%, and 8.3% of the 360 respondents have rated the quality of the learning experience as excellent, good and satisfactory, respectively (RR = 53.3%). We have regularly incorporated feedback from students about changes that could improve the course. This included giving them further guidance about their assessment task, use of additional pre-tutorial resources, and discussion of optional activities that students could be involved with including the Australian Medical Students' Association (AMSA) Code Green initiative and attending a local “green” operating theater that featured environmentally sustainable innovations. A number of students have taken advantage of this opportunity. Two publications spotlight an example of how our course inspired further scholarship and student advocacy in the area of environmental sustainability in anesthetic practice (54, 55).

## 5 Discussion

Medical school curricula need to feature the changing environmental determinants of health and how students and doctors can be agents of positive change. However, the principle of “first do no harm” applies to the learning environment as it does to healthcare. How do we care for our students as they learn about planetary health, when increased awareness of climate change and other ecosystem changes can lead to distress, poorer mental health and burnout especially amongst those who may already be involved in advocacy?

We have applied several principles from motivational psychology in how we have delivered our sustainable health course, but more can be done. Educators need to be mindful of the impacts of planetary health content and assist learners in coming to terms with the enormity of the challenge and showing a path toward progress. Acceptance and commitment therapy or training, offers the practice of avoiding being hooked by unhelpful thoughts and ruminations that can lead to despair and instead identifying productive ways of thinking about the opportunities for moving in desired directions. Committed action is best planned by first being in touch with one's values. Our students are invited to consider their values in thinking about how health professional advocacy could achieve progress toward a sustainable developmental goal in a particular group in one exercise. However, this principle could be further applied by incorporating subsequent projects or teamwork exercises.

Self -determination theory explains that people are more likely to maintain intrinsic motivation and enjoyment in learning if their environment promotes autonomy, competence and relatedness. In our course we have implemented a number of strategies to enhance the learner's autonomy. Students chose which discussion points to lead during the tutorials, and the direction in which they take these. Students also have autonomy over the areas of interest to research and present to the group. The self-paced modular content and tutorials on zoom also provide flexibility and convenience for students. There are however limitations on autonomy in that the course and assessment are compulsory. This is overcome by some programs in having environmental sustainability as an elective choice, however this limits the reach of this important area of curriculum development.

In terms of relatedness, students in our example learn in small groups with a dedicated tutor, and have the opportunity to consider each other's perspectives as they reflect on their own learning and personal experiences. The reflective task in module one which asks students to describe what climate change impacts they have observed where they live or within the location they call home, fosters connection within the group by recognition of common experiences and concerns. A sense of relating to their community of practice is fostered by incorporating presentations from medical leaders who display openness in sharing their own challenges, hopes and fears. Likewise, the host in the featured mainstream media production on the carbon footprint of foods, shows vulnerability by expressing his own concerns, knowledge gaps and personal mistakes (56). Finally, the assessment task is a time for students to learn from each other and provides the opportunity for shared learning and encouragement. Further possibilities to strengthen a sense of relatedness could include increasing time allocated within the course for group activities and sustainability projects with their wider community of practice.

Students may develop a sense of competence by problem-solving clinical approaches to a variety of patient presentations that relate to climate change and environmental health impacts. The design of the assignment allows students to acquire depth of knowledge in their particular areas of interest, which they utilize to educate and facilitate discussion with their peers. A number of the reflective tasks also provide students with an opportunity to practice a new skill and receive group feedback. In module 2, for example, students reflect on the coaching model of balancing support and challenge, and describe how they could utilize this to make a change in their own lives. Students bring this to the tutorial to discuss and receive feedback. While these tasks support the development of competence, students are not formally assessed on levels of competence during the course. Ways of doing so could include embedding hands-on experiences within the course for students to practice and be assessed on their clinical or advocacy skills.

Our course draws on concepts of adaptive coping. Within the first week of our course we check in with students during the tutorial about their reactions to the content and provide them with resources that promote emotion focused coping (39). Students are invited to share particular adaptive coping strategies that resonate with them. Part of our strategy is to provide positive examples of progress in environmental sustainability, and provide a sense of hope for a better future. A benefit of this is that students learn about skills that could help their own future patients to deal with climate change distress.

Educators in all disciplines need to consider how best to engage learners. Planetary health has the potential to overwhelm learners with its scope, and to lead to distress about the concerning trajectory of climate change and environmental degradation. At the same time, there is the opportunity to inspire medical students to embrace the challenge of protecting the health of patients and communities from current impacts of extreme weather events and ecosystem damage, and to develop adaptive capacity for a changing world. Being mindful of relevant and helpful psychological theories and interventions can help to sustain learners and to assist in positive change and solutions that promote environmental sustainability and safeguard the future.

## 6 Acknowledgment of context

Introducing new curricula with considerable time allocation is typically challenging due to the existing content-dense medical curricula. In this case, the cancellation of an elective community placement in which many students traveled overseas, due to the COVID-19 pandemic provided an opportunity to introduce this content. Students now have the opportunity to learn about planetary health embedded into their medical education, while the overseas elective opportunities were shifted to later in the degree.

## Data Availability

The original contributions presented in the study are included in the article/supplementary material, further inquiries can be directed to the corresponding author.
